# Treatment of Multiple Myeloma in Patients Refractory to Daratumumab/Anti‐CD38 Monoclonal Antibodies: A Systematic Review

**DOI:** 10.1002/cam4.70585

**Published:** 2025-03-07

**Authors:** Chia Jie Tan, Dylan Kacerek, Nattawara Kampirapawong, Amandeep Godara, Nathorn Chaiyakunapruk

**Affiliations:** ^1^ Department of Pharmacotherapy, College of Pharmacy University of Utah Salt Lake City Utah USA; ^2^ University of Phayao Phayao Thailand; ^3^ Division of Hematology and Hematologic Malignancies, Huntsman Cancer Institute University of Utah Salt Lake City Utah USA; ^4^ IDEAS Center, Veterans Affairs Salt Lake City Healthcare System Salt Lake City Utah USA

**Keywords:** daratumumab, multiple myeloma, refractory, relapsed, systematic review

## Abstract

**Background:**

There is a lack of clear guidance on the appropriate next choice of therapy for patients with relapsed/refractory multiple myeloma who become refractory to daratumumab, an anti‐CD38 antibody. This review aims to identify and compare treatments with published clinical trial evidence among patients with daratumumab‐refractory multiple myeloma.

**Methods:**

MEDLINE, Cochrane CENTRAL, and EMBASE databases were searched for clinical trials that evaluated treatments for patients with multiple myeloma who were refractory to daratumumab from November 2015 to October 2023. Eligible studies may have enrolled only patients who were refractory to daratumumab or reported findings on patients with daratumumab‐refractory disease in subgroup analyses. Treatment outcomes of interest included response rates and survival outcomes. Screening and data extraction were done independently by two reviewers using covidence, and any discrepancy was resolved by a third reviewer. Qualitative synthesis was performed to describe and compare patient outcomes associated with different treatments.

**Results:**

A total of 33 papers/published studies, representing 23 clinical trials, were eligible/included. Interventions from the eligible trials include chimeric antigen receptor (CAR)‐T cell therapy, B‐cell maturation antigen (BCMA)‐directed antibodies, other monoclonal antibodies, cereblon E3 ligase modulators, a peptide‐drug conjugate, and other targeted therapies. CAR T‐cell therapy demonstrated the highest overall response rates, longer median overall, and progression‐free survival in addition to significantly lower risk of death and higher odds of response compared to standard of care. Similarly, high response rates and/or long‐term survival was also observed for other BCMA‐directed treatments, such as elranatamab and teclistamab.

**Conclusion:**

Based on the results of this systematic review, BCMA‐directed therapies such as CAR‐T cell therapy and bispecific antibodies demonstrate promising efficacy among patients with anti‐CD38 refractory disease. However, additional evidence from randomized clinical trials is necessary to establish best practice guidelines.

## 1 Introduction

Based on cancer registry data from 2014 to 2020, the Surveillance, Epidemiology, and End Results Program estimates a 5‐year survival rate of 61.1% for patients with multiple myeloma [1]. However, given the natural history of multiple myeloma, disease relapse is common and most patients undergo multiple cycles of treatment. While autologous stem cell transplant remains a cornerstone in multiple myeloma treatment, the peak incidence of multiple myeloma is among individuals older than 65 years old and a substantial proportion of patients are not transplant candidates due to age and comorbidities [2]. Other treatment options for multiple myeloma are cytotoxic chemotherapy, corticosteroids, immunomodulatory drugs (IMiDs), proteasome inhibitors (PI), nuclear export inhibitors, monoclonal antibodies (such as antibody drug conjugates [ADCs], and bispecific T‐cell engagers [BiTEs] targeting a wide range of proteins including CD38) and chimeric antigen receptor (CAR‐T) cell therapy [2]. As treatment through multiple disease relapses requires use of drug combinations, there is an ongoing demand for new therapeutic agents, particularly for patients with relapsed and/or refractory multiple myeloma (RRMM).

Daratumumab, an anti‐CD38 monoclonal antibody, since its incorporation into the treatment paradigm for newly diagnosed multiple myeloma has enhanced overall survival (OS) and progression‐free survival (PFS) in both transplant eligible and ineligible patients [3, 4, 5]. Daratumumab was originally approved by the Food and Drug Administration (FDA) in 2015 for multiple myeloma patients who had previously attempted a PI and an IMiD [6]. Based on evidence from the ALCYONE, CASSIOPEIA, and MAIA trials, daratumumab's labeled indication has now been expanded to the first line setting for newly diagnosed patients, irrespective of transplant eligibility [7, 8, 9]. Combination therapies with daratumumab have a clinical response rate of 67%–81% indicating drug synergy as daratumumab monotherapy only has a response rate of around 31% [10]. Expert panel guidelines are available for RRMM patients who have previously received bortezomib and lenalidomide [11]. However, there is a need to synthesize published clinical data on the treatment response and outcomes of daratumumab‐refractory patients, considering the growing numbers of daratumumab‐refractory patients that are also likely to be refractory to multiple other agents.

The aim of the current study is to conduct a systematic literature review to summarize the efficacy of treatments for patients with multiple myeloma and who are refractory to daratumumab. This literature review will contribute to evidence‐based recommendations for patients with multiple myeloma to assist clinicians in making treatment decisions for patients who are refractory to daratumumab.

## 2 Methods

A systematic review was conducted in accordance with the Cochrane Handbook for Systematic Reviews of Interventions and reported according to the PRISMA guidelines [12, 13]. Details of the protocol for this review were registered on PROSPERO and can be accessed at www.crd.york.ac.uk/PROSPERO/display_record.asp?ID=CRD42024571990. Deviations from the protocol are reported in Supporting Information S1.

### 2.1 Search Strategy

MEDLINE, Cochrane CENTRAL, and EMBASE databases were searched for studies evaluating treatments for RRMM from November 2015 until October 2023. Search terms included Medical Subject Headings and associated keywords for “multiple myeloma,” “refractory,” and “relapse.” The search included only papers published since November 2015, which was when daratumumab was first approved by the FDA in the United States. No language restriction was applied and validated search filters were used to identify only clinical trials. The complete search syntax for each database is shown in Supporting Information S2. To capture studies that may have been missed, citation searching was performed and the final list of eligible papers was reviewed by a clinician specialized in multiple myeloma treatment (A.G.).

### 2.2 Study Selection

Any clinical trial that evaluated treatments for multiple myeloma and reported treatment outcomes of patients who were refractory to daratumumab were eligible for this review. For this review, a clinical trial was defined as a research study where human subjects were prospectively assigned to an intervention to assess the effect of interventions on health‐related outcomes. Trials were included regardless of clinical trial phase, whether a control arm was present, or whether randomization was conducted. Adopting the conventional definition in multiple myeloma trials, patients were considered to be refractory to daratumumab if progressive disease or stable disease was the best response to daratumumab in a prior line of therapy and/or progressive disease was observed within 60 days after completion of treatment. Exposure to daratumumab alone was inadequate to meet the criteria for refractoriness to daratumumab. Trials that were eligible may have enrolled only patients who were refractory to daratumumab or reported findings from a subgroup of patients who were refractory to daratumumab. Findings may be reported for patients who were refractory specifically to daratumumab or in combination with other drugs, for example, all CD38‐directed monoclonal antibodies, in addition to a PI and an IMiD (triple‐refractory) or in addition to two PIs and two IMiDs (penta‐refractory). Treatment outcomes should be reported as treatment response rates and/or survival outcomes. Systematic reviews, editorials, commentaries, and observational studies were not eligible. Conference abstracts and short correspondences were included if sufficient data were available to determine eligibility and outcomes of interest were adequately reported.

Covidence was used for study screening. After deduplication of the search results from the three databases, two reviewers (D.K. and N.K.) independently screened titles and abstracts to determine if papers potentially met the eligibility criteria. Disagreements were resolved by a third reviewer (C.J.T.). The full texts of all potentially eligible papers were obtained, and the same process was repeated to determine papers that fully met the eligibility criteria. No automated tools were used in the screening process. Studies that appeared to meet the eligibility criteria but were excluded are shown in Supporting Information S3. Papers reporting on the same trial were identified based on clinical trial registration numbers or trial names. A study selection flow diagram was generated using an online digital tool compliant to the PRISMA guidelines [14].

### 2.3 Data Extraction

Data from eligible studies were independently extracted by two reviewers (D.K. and N.K.) using a pre‐piloted form on Covidence and discrepancies were resolved by a third reviewer (C.J.T.). Data elements that were collected included study characteristics, key eligibility criteria, intervention characteristics, patient demographics, and treatment outcomes. For studies in which patients with daratumumab‐refractory disease were reported in a subgroup analysis and patient demographics of the subgroup were unavailable, characteristics of the full patient cohort were extracted instead. Treatment outcomes of interest included response rates and survival outcomes of patients with daratumumab‐refractory disease. Response rates referred to the proportion of patients who met the criteria for treatment response categories outlined in the International Myeloma Working Group Uniform Response Criteria for Multiple Myeloma [15]. The overall response rate (ORR) included patients who had achieved complete response, very good partial response and partial response. Survival outcomes referred to median OS and PFS. In studies with more than one study arm, the relevant odds and hazard ratios of treatment response and survival, respectively, were abstracted if available. If similar outcomes were reported at different timepoints or as overlapping subgroups, findings from the latest time point or largest subgroup were included in our review.

### 2.4 Data Analysis and Quality Assessment

The interventions were grouped into four main therapeutic classes: CAR‐T cell therapy, B‐cell maturation antigen (BCMA)‐directed monoclonal antibodies, non‐BCMA‐directed monoclonal antibodies, and others. Due to the heterogeneity in study population, no quantitative synthesis was conducted. The treatment response rates and survival outcomes for each drug and therapeutic class were tabulated and compared qualitatively.

The quality of randomized controlled trials was assessed using the Cochrane Risk of Bias 2 tool (ROB2), which encompasses five key domains related to bias arising from (i) randomization, (ii) deviations from intended intervention, (iii) missing outcomes, (iv) outcome measurement, and (v) selection of reported results [16]. For other study designs, the Risk of Bias In Non‐randomized Studies of Interventions tool (ROBINS‐I) was employed, which included seven key domains related to bias arising from: (i) confounding, (ii) selection of participants, (iii) classification of interventions, (iv) deviation from intended intervention, (v) missing data, (vi) outcome measurement, and (vii) selection of reported results. In studies without a comparator arm (such as single‐arm studies), the individual domains were evaluated by considering if bias in each individual domain can be addressed if an external control arm was available [17]. For both ROB2 and ROBINS‐I, quality assessment was performed by domain, and an overall level was determined based on the risk of bias in each domain. Traffic light and summary plots were generated using the *robvis* web application [18].

## 3 Results

### 3.1 Study Selection

**FIGURE 1:**
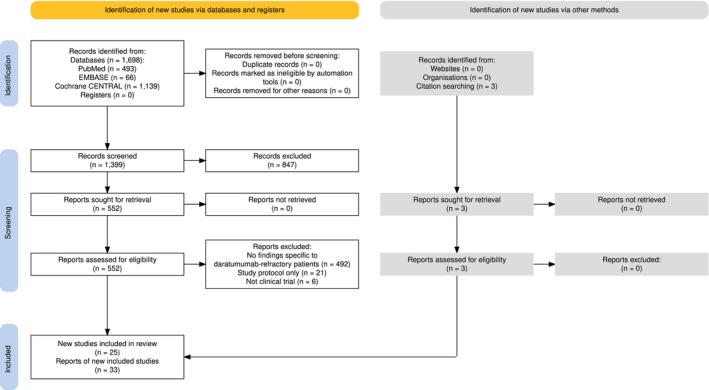
PRISMA flowchart of included studies.

Across the three databases, 1698 papers were identified, leaving 1399 papers to be initially screened based on titles and abstracts after duplicate publications were removed (Figure 1). A total of 552 papers underwent full text review and 519 were excluded. Together with 3 papers identified by citation searching, a total of 33 papers, representing data from 25 studies, were found to be eligible for inclusion (Table 1).

**TABLE 1:** Study characteristics of included studies stratified by therapeutic class of intervention.

Trial^a^	Reports	Study design (phase)	Location enrollment period	Intervention	Key eligibility criteria				
					PriorLOT	PI	IMiD	CD38 AB	ECOG PS
CAR‐T cell therapy									
NCT04155749 [19]	Frigault 2023	Single arm (I)	United States 11/2019 to 04/2021	CART‐ddBCMA (anti‐BCMA CART)	≥ 3	R	R	R	0/1
NCT04720313 [20]	Asherie 2023	Single arm (I)	Israel 01/2021 to 12/2021	HBI0101 (anti‐BCMA CART)	≥ 3	E	E	E^b^	NS
UNIVERSAL [21]	Mailankody 2023	Single arm (I)	United States 09/2019 to 10/2021	ALLO‐715 (anti‐BCMA CART)	≥ 3	E	E	E	0/1
CARTITUDE‐1 [22]	Martin 2022	Single arm (I/II)	International 06/2018 to 07/2019	Cilta‐cel	≥ 3	R	R	E	0/1
CARTITUDE‐4 [23]	San‐Miguel 2023	Randomized controlled trial (III)	International 07/2020 to 11/2021	Cilta‐cel vs. DPd/PVd	1–3	E	R^c^	NS	0/1
KarMMa [24, 25]	Munshi 2021 Minakata 2023	Single arm (II)	International 12/2017 to 11/2018	Ide‐cel	≥ 3	E	E	E	0/1/2
KarMMa‐3 [26]	Rodriguez‐Otero 2023	Randomized controlled trial (III)	International 05/2019 to 04/2022	Ide‐cel vs. DPd/DVd/EPd/IRd/Kd	2–4	E	E	E	0/1
BCMA‐directed monoclonal antibodies (including ADCs and BiTEs)									
DREAMM‐2 [27, 28, 29, 30]	Richardson 2020 Lonial 2020 Lonial 2021 Ramasamy 2023	Randomized dose comparison trial (II)	International 06/2018 to 01/2019	Belantamab mafodotin	≥ 3	R	R	R/I	0/1/2
MagnetisMM‐3 [31]	Lesokhin 2023	Single arm (II)	International 02/2021 to 01/2022	Elranatamab	NS	R	R	R	0/1/2
NCT03933735 [32]	D'Souza 2022	Single arm (I)	International 06/2019 to 01/2022	ABBV‐383 (anti‐BCMA BiTE)	≥ 3	E	E	E	0/1/2
NCT02561962 [33]	Lee 2021	Single arm (I)	International 11/2015 to 06/2018	**AMG 224 (anti‐BCMA ADC)**	≥ 3	E	E	NS	0/1/2
MajesTEC‐1 [34, 35]	Usmani 2021 Moreau 2022	Single arm (I; II)	International 03/2020 to 08/2021	Teclistamab	≥ 3	E	E	E	0/1
Non‐BCMA‐directed monoclonal antibodies (including ADCs and BiTEs)									
NCT03713294 [36]	Ailawadhi 2022	Single arm (II)	United States NR	Elotuzumab + Pomalidomide	≥ 1	NS	NS	R	0/1/2
NCT02514668 [37]	Mikhael 2021	Single arm (II)	International NR	Isatuximab	NS	E	E	R	NS
NCT02283775 [38]	Usmani 2021	Single arm (I)	United States 03/2018 to 12/2018	Isatuximab + Pomalidomide	≥ 2	E	E	NS	0/1/2
MonumenTAL‐1 [39]	Chari 2022	Single arm (I)	International 01/2018 to 11/2021	Talquetamab	NS	E	E	NS	0/1
TRIMM‐2 [40]	Dholaria 2023	Single (I)	International 02/2020 to NR	Talquetamab + Datatumumab	≥ 3^d^	E	E	NS^d^	0/1
Others									
CC‐220‐MM‐001 [41, 42]	Lonial 2021 Lonial 2022	Single arm (I; II)	International 12/2016 to 03/2020	Iberdomide	≥ 3	R	R	R	0/1/2
NCT03374085 [43]	Richardson 2023	Single arm (I; II)	International 02/2018 to 09/2022	Mezigdomide	≥ 3	R	R	R	0/1/2
STORM [44, 45, 46]	Vogl 2016 Vogl 2018 Chari 2019	Single arm (II)	International 05/2015 to 03/2018	Selinexor	≥ 3	R	R	R	0/1/2
HORIZON [47]	Richardson 2021	Single arm (II)	International 12/2016 to 10/2019	**Melflufen**	≥ 2	E	E	R^e^	0/1/2
OCEAN [48]	Dimopoulos 2021	Randomized controlled trial (III)	International 06/2017 to 09/2020	**Melflufen** vs. pomalidomide	2–4	E	R	NS	0/1/2
STOMP [49]	Gasparetto 2022	Single arm (I)	International 03/2018 to 02/2021	Selinexor + Carfilzomib	≥ 1	NS	NS	NS	0/1/2
MARCH [50]	Qiu 2022	Single arm (II)	China 08/2019 to 02/2021	Selinexor	NS	E	E	E	0/1/2
NCT02899052 [51]	Costa 2021	Single arm (II)	United States 02/2017 to 02/2019	Venetoclax + Carfilzomib	1–3	NS	NS	NS	0/1/2

*Note:* Data of patients with daratumumab‐refractory disease were obtained from subgroup analyses for studies in shaded rows. Drugs in bold have been withdrawn from the market or developmental pipeline.

Abbreviations: ADC, antibody‐drug conjugate; BCMA, B‐cell maturation antigen; BiTE, bispecific T‐cell engager; CART, chimeric antigen receptor‐T cell therapy; CD38 AB, CD38‐directed antibodies; DPd, daratumumab + pomalidomide + dexamethasone; DVd, daratumumab + bortezimib + dexamethasone; E, prior exposure; ECOG PS, Eastern Cooperative Oncology Group Performance Status; EPd, elotuzumab + pomalidomide + dexamethasone; I, prior intolerance; IMiD, immunomodulators; IRd, ixazomib + lenalidomide + dexamethasone; Kd, carfilzomib + dexamethasone; LOT, lines of therapy; NS, not specified; PI, protease inhibitors; PVd, pomalidomide + bortezomib + dexamethasone; R, prior refractoriness.

^a^ Trial names were based on those used in publications or trial registries. If unavailable, trial registration numbers on ClinicalTrials.gov were used.

^b^ After enrollment, all participants were found to be refractory to daratumumab although the eligibility criteria only specified prior exposure to daratumumab.

^c^ Only to lenalidomide.

^d^ Alternatively, patients were eligible if presenting with double‐refractory disease. Patients with exposure to anti‐CD38 therapy in the 90 days prior to enrollment or any history of intolerance were excluded.

^e^ Refractory o CD38‐directed monoclonal antibodies or pomalidomide.

### 3.2 Study Characteristics

Among the 25 studies, all participants in 9 studies (36.0%) were refractory to daratumumab, primarily as the inclusion criteria mandated enrollment of patients who were refractory to daratumumab or CD38‐directed therapy [19, 20, 27, 28, 29, 30, 31, 36, 37, 41, 42, 43, 44, 45, 46]. In the remaining 16 studies (64.0%), data of patients with daratumumab‐refractory disease were presented in subgroup analyses [21, 22, 23, 24, 25, 26, 32, 33, 34, 35, 38, 39, 40, 47, 48, 49, 50, 51]. The characteristics of the eligible studies are presented in Table 1. The interventions of the eligible studies included CAR T‐cell therapy (CART‐ddBCMA, HB10101, ALLO‐715, cilta‐cel, and ide‐cel); BCMA‐directed ADCs (belantamab and AMG 224); BCMA‐directed BiTEs (elranatamab, ABBV‐383 and teclistamab); non‐BCMA‐directed monoclonal antibodies (elotuzumab, isatuximab, and talquetamab); cereblon E3 ligase modulators (iberdomide and mezigdomide); a peptide‐drug conjugate (melflufen); and other targeted therapies (selinexor and venetoclax). Most studies were in phase I or II (*n* = 22, 88.0%) [19, 20, 21, 22, 24, 25, 27, 28, 29, 30, 31, 32, 34, 35, 36, 37, 38, 39, 40, 41, 42, 43, 44, 45, 46, 47, 49, 50, 51] to identify the maximum tolerated dose and/or generate preliminary safety and efficacy data while three trials (13.7%), CARTITUDE‐4, KarMMa‐3, and OCEAN [23, 26, 48], were randomized controlled trials to evaluate the efficacy and safety of new agents against the standard of care. All phase I and II trials were single‐arm studies with the exception of DREAMM‐2 [27, 28, 29, 30], which was a randomized two‐arm study comparing different doses of belantamab. Half of the studies (*n* = 13, 52.0%) required participants to have had at least three lines of prior therapy, including a PI, an IMiD, and/or a CD38‐directed antibody [19, 20, 21, 22, 24, 25, 27, 28, 29, 30, 32, 33, 34, 35, 40, 41, 42, 43, 44, 45, 46].

The characteristics of participants in the eligible studies are shown in Supporting Information S4 and largely reflected the demographics of patients with RRMM. Briefly, the median age was between 60 and 73 years old, with a trend of younger participants enrolled in studies examining CAR T‐cell therapy compared to other types of treatment. A slightly higher proportion of males were observed across all studies except for 2 (NCT04720313 and NCT02561962) [20, 33]. As expected for clinical trials, most patients in the eligible studies had an ECOG performance status of 0 or 1 at enrollment. The proportion of patients with high‐risk cytogenetics ranged widely from 12% to 90%. The median number of lines of prior therapy also varied considerably from 1 to 7, with a large proportion of patients documented to have had prior exposure or were refractory to PIs and/or IMiDs.

### 3.3 Treatment Response

ORR was reported for patients with daratumumab‐refractory disease in almost all eligible studies, except for OCEAN (melflufen), and CARTITUDE‐4 (cilta‐cel) (Table 2). The highest ORRs were consistently observed among patients receiving CAR‐T cell therapy. NCT04155749, CARTITUDE‐1, and NCT04720313 reported the highest ORRs at 100% for CART‐ddBCMA, 98% for cilta‐cel, and 75% for HB10101 (all anti‐BCMA CAR‐T cell therapy), respectively, with the caveat that only a small number of patients were analyzed in two of the three studies [19, 20, 22]. Considering only larger studies with at least 100 participants, another anti‐BCMA CAR‐T cell therapy ide‐cel showed the highest ORR at 72% in phase II KarMMa and 71% in phase III KarMMa‐3, respectively [24, 26]. Notably in KarMMa‐3, patients who received ide‐cel had 3.5 higher odds (95% CI: 2.2–5.5) of achieving a response compared to those in the control arm [26]. Several monoclonal antibodies also demonstrated an ORR of > 50%, including teclistamab (63% in MajesTEC‐1) [35], elranatamab (61% in MagnetisMM‐3) [31], ABBV‐383, an anti‐BCMA BiTE (54% in NCT03933735) [32] and talquetamab (76% in TRIMM‐2, 65%–70% in MonumenTAL‐1) [39, 40]. Except for TRIMM‐2, these response rates were reported among patients with triple‐refractory disease and all but talquetamab were BCMA‐directed agents. Selinexor together with carfilzomib showed an ORR of 67% in STOMP [49]; however, other trials evaluating selinexor alone (STORM and MARCH) yielded ORRs of 26% and 25%, respectively [46, 50]. Elotuzumab demonstrated a response rate of 35% in combination with pomalidomide while isatuximab, another CD38‐directed antibody, had poor response rates at 0% when used alone in NCT02514668 and 14% with pomalidomide in NCT02283775 [36, 37, 38]. A detailed distribution of patients who achieved complete response, very good partial response and partial response were available for a few studies and largely followed the trends of ORR.

**TABLE 2:** Treatment response from included studies stratified by therapeutic class of intervention.

Trial	Intervention	Refractory status^a^	*N* analyzed	Response rates				mTTR in months (range/95% CI)	mDOR in months (range/95% CI)
				ORR	CR	VGPR	PR		
CAR‐T cell therapy									
NCT04155749 [19]	CART‐ddBCMA (anti‐BCMA CART)	Triple	12	100%	75%	8%	17%	0.9 (0.9–1.9)	NR
NCT04720313 [20]	HBI0101 (anti‐BCMA CART)	Triple	22	75%	50%	25%	0%	NR	NR
UNIVERSAL [21]	ALLO‐715 (anti‐BCMA CART)	Penta	9	44%	NR	NR	NR	NR	NR
CARTITUDE‐1 [22]	Cilta‐cel	Triple	85	98%	NR	NR	NR	NR	NE (24.3 to NE)
CARTITUDE‐4 [23]	Cilta‐cel SoC (DPd/PVd)	CD38 AB	48 45	NR	NR	NR	NR	NR	NR
KarMMa [24, 25]	Ide‐cel	Triple	108	72%	NR	NR	NR	NR	NR
KarMMa‐3 [26]	Ide‐cel SoC (DPd/DVd/EPd/IRd/Kd)	Daratumumab	242 123	71% 42% OR: 3.5 (2.2–5.5)	NR	NR	NR	NR	NR
BCMA‐directed monoclonal antibodies (including ADCs and BiTEs)									
DREAMM‐2 [27, 28, 29, 30]	Belantamab mafodotin 2.5 mg/kg 3.4 mg/kg	CD38 AB	97 99	32% 35%	19% 24%		12% 11%	1.5 (NR) 1.4 (NR)	12.5 (NR) 6.2 (NR)
MagnetisMM‐3 [31]	Elranatamab	Triple	123	61%	35%	21%	5%	1.2 (0.9–7.4)	NR
NCT03933735 [32]	ABBV‐383 (anti‐BCMA BiTE)	Triple	41	54%	24%	12%	17%	NR	NR
NCT02561962 [33]	**AMG 224 (anti‐BCMA ADC)**	Daratumumab	7	14%	NR	NR	NR	NR	NR
MajesTEC‐1 [34, 35]	Teclistamab	Triple	128	63%	NR	NR	NR	NR	NR
Non‐BCMA‐directed monoclonal antibodies (including ADCs and BiTEs)									
NCT03713294 [36]	Elotuzumab (+ Pom)	Daratumumab	37	35%	0%	3%	32%	2.9	NR
NCT02514668 [37]	Isatuximab	Daratumumab	32	0%	0%	0%	0%	NR	NR
NCT02283775 [38]	Isatuximab (+ Pom)	Daratumumab	7	14%	0%	0%	14%	NR	NR
MonumenTAL‐1 [39]	Talquetamab 405 mcg/kg 800 mcg/mL	Triple	23 33	65% 70%	NR	NR	NR	NR	NR
TRIMM‐2 [40]	Talquetamab + Daratumumab	Anti‐CD38	50	76%	NR	NR	NR	NR	NR
Others									
CC‐220‐MM‐001 [41, 42]	Iberdomide	CD38 AB	107	26%	1%	8%	17%	1.0 (0.9–2.5)	7.0 (4.5–11.3)
NCT03374085 [43]	Mezigdomide	Triple	101	41%	5%	20%	16%	NR	7.6 (5.4–9.5)
STORM [44, 45, 46]	Selinexor	Triple	122	26%	2%	5%	20%	0.9 (0.2 to 3.2)	4.4 (3.7–10.8)
HORIZON [47]	**Melflufen** SoC (Pom)	CD38 AB	48 39	NR	NR	NR	NR	NR	NR
OCEAN [48]	**Melflufen**	Triple	119	26%	0%	11%	15%	1.9 (1.0–6.1)	4.4 (3.4–7.6)
STOMP [49]	Selinexor (+ Car)	Triple	12	67%	0%	50%	17%	NR	22.7 (13.1‐NE)
MARCH [50]	Selinexor	Triple	20	25%	NR	NR	NR	NR	NR
NCT02899052 [51]	Venetoclax (+ Car)	Daratumumab	1	0%	0%	0%	0%	NR	NR

*Note:* All patients were refractory to daratumumab for studies in unshaded rows while patients with daratumumab‐refractory disease were analyzed in subgroup analyses for studies in shaded rows. Drugs in bold have been withdrawn from the market or developmental pipeline.

Abbreviations: ADC, antibody‐drug conjugate; BCMA, B‐cell maturation antigen; BiTE, bispecific T‐cell engager; CART, chimeric antigen receptor‐T cell therapy; Car, carfilzomib; CD38 AB, CD38‐directed antibodies; CI, confidence interval; CR, complete response; Dex, dexamethasone; DPd, daratumumab + pomalidomide + dexamethasone; DVd, daratumumab + bortezimib + dexamethasone; EPd, elotuzumab + pomalidomide + dexamethasone; IRd, ixazomib + lenalidomide + dexamethasone; Kd, carfilzomib + dexamethasone; mDOR, median duration of response; mTTR, median time to response; NE, not evaluable; NR, not reported; OR, odds ratio; ORR, overall response rate; Pom, pomalidomide; PR, partial response; PVd, pomalidomide + bortezomib + dexamethasone; VGPR, very good partial response.

^a^ Indicates if patients analyzed were refractory to only daratumumab, refractory to all CD38‐directed antibodies, triple‐refractory or penta‐refractory.

### 3.4 Survival Outcomes

A total of 14 studies reported at least one survival outcome with a median duration of follow‐up ranging between 3.7 and 27.7 months (Table 3). Compared to other therapeutic classes, CAR‐T cell therapy reported the longest PFS. At a median duration of follow‐up of 12.6 months, the median PFS was not reached for CART‐ddBCMA (an anti‐BCMA CAR‐T cell therapy) while ide‐cel demonstrated a median PFS of 12.5 months (95% CI: 11.3–15.1) [19, 26]. The HRs reported for cilta‐cel and ide‐cel indicated superiority of CAR‐T cell therapy over the control arm in terms of PFS at 0.26 (95% CI: 0.14–0.50) and 0.51 (0.39–0.67), respectively [23, 26]. The median OS was not reached for CART‐ddBCMA (an anti‐BCMA CAR‐T cell therapy) and 10.1 months for HB10101 (an anti‐BCMA CAR‐T cell therapy) [19, 20]. OS data for other CAR‐T cell therapies were not reported.

**TABLE 3:** Survival outcomes from included studies stratified by therapeutic class.

Trial	Intervention	Refractory status^a^	*N* analyzed	Median duration of follow‐up in months (range)	mPFS in months (95% CI)	mOS in months (95% CI)
CAR‐T cell therapy						
NCT04155749 [19]	CART‐ddBCMA (anti‐BCMA CART)	Triple	12	12.6	Not reached	Not reached
NCT04720313 [20]	HBI0101 (anti‐BCMA CART)	Triple	22	4.5	5.2	10.1
UNIVERSAL [21]	ALLO‐715 (anti‐BCMA CART)	Penta	9	10.2 (3.8 to NE)	NR	NR
CARTITUDE‐1 [22]	Cilta‐cel	Triple	85	27.7 (NR)	Not reached (25.2–NE)	69.7 (58.4–78.5)
CARTITUDE‐4 [23]	Cilta‐cel SoC (DPd/PVd)	CD38 AB	48 45	15.9 (0.1 to 27.3)	NR HR: 0.26 (0.14–0.50)	NR
KarMMa [24, 25]	Ide‐cel	Triple	108	13.3 (0.2–21.2)	NR	NR
KarMMa‐3 [26]	Ide‐cel SoC (DPd/DVd/EPd/IRd/Kd)	Daratumumab	242 123	18.6 (0.4–35.4)	12.5 (11.3–15.1) 4.4 (3.2–5.9) HR: 0.51 (0.39–0.67)	NR
BCMA‐directed monoclonal antibodies (including ADCs and BiTEs)						
DREAMM‐2 [27, 28, 29, 30]	Belantamab mafodotin 2.5 mg/kg 3.4 mg/kg	CD38 AB	97 99	12.5 13.8	2.8 3.9	15.3 14.0
MagnetisMM‐3 [31]	Elranatamab	Triple	123	14.7 (0.2 to 25.1)	Not reached (9.9–NE)	Not reached (13.9–NE)
NCT03933735 [32]	ABBV‐383 (anti‐BCMA BiTE)	Triple	41	8.2 (0.6–11.5)	NR	NR
NCT02561962 [33]	**AMG 224 (anti‐BCMA ADC)**	Daratumumab	7	NR	NR	NR
MajesTEC‐1 [34, 35]	Teclistamab	Triple	128	14.1 (0.3–24.4)	NR	NR
Non‐BCMA‐directed monoclonal antibodies (including ADCs and BiTEs)						
NCT03713294 [36]	Elotuzumab (+ Pom)	Daratumumab	37	23.4 (14.6–37.2)	3.7 (2.9–11.1)	NR
NCT02514668 [37]	Isatuximab	Daratumumab	32	1.9 (0.8–17)	1.6 (1.0–3.2)	10.7 (8.0–19.0)
NCT02283775 [38]	Isatuximab (+ Pom)	Daratumumab	7	9.9 (0–17.3)	NR	NR
MonumenTAL‐1 [39]	Talquetamab 405 mcg/kg 800 mcg/mL	Triple	23 33	11.7 (1.0–21.2) 4.2 (0.7–13.7)	NR	NR
TRIMM‐2 [40]	Talquetamab + Daratumumab	Anti‐CD38	50	NR	NR	NR
Others						
CC‐220‐MM‐001 [41, 42]	Iberdomide	CD38 AB	107	7.7 (5.3–11.4)	3.0 (2.8–3.7)	10.7 (8.8–NE)
NCT03374085 [43]	Mezigdomide	Triple	101	7.5 (0.5–21.9)	4.4 (3.0–5.5)	NR
STORM [44, 45, 46]	Selinexor	Triple	122	NR	3.7 (3.0–5.3)	8.6 (6.2–11.3)
HORIZON [47]	**Melflufen** SoC (Pom)	CD38 AB	48 39	15.5 (9.4–22.8) 16.3 (10.1–23.2)	Median NR HR: 1.05 (0.64–1.73)	Median NR HR: 1.62 (0.82–3.21)
OCEAN [48]	**Melflufen**	Triple	119	14 (10.8–18.7)	3.9 (3.0–4.6)	11.2 (7.7–13.2) 1 year EFS: 42%
STOMP [49]	Selinexor (+ Car)	Triple	12	15.1	23.7 (3.9–NE)	20.4 (20.4–NE)
MARCH [50]	Selinexor	Triple	20	10.6	2.9 (1.65–5.62)	11.9 (2.0–NE)
NCT02899052 [51]	Venetoclax (+ Car)	Daratumumab	1	NR	NR	NR

*Note:* All patients were refractory to daratumumab for studies in unshaded rows while patients with daratumumab‐refractory disease were analyzed in subgroup analyses for studies in shaded rows. Drugs in bold have been withdrawn from the market or developmental pipeline.

Abbreviations: ADC, antibody‐drug conjugate; BCMA, B‐cell maturation antigen; BiTE, bispecific T‐cell engager; CART, chimeric antigen receptor‐T cell therapy; Car, carfilzomib; CD38 AB, CD38‐directed antibodies; CI, confidence interval; Dex, dexamethasone; DPd, daratumumab + pomalidomide + dexamethasone; DVd, daratumumab + bortezimib + dexamethasone; EPd, elotuzumab + pomalidomide + dexamethasone; HR, hazards ratio; IRd, ixazomib + lenalidomide + dexamethasone; Kd, carfilzomib + dexamethasone; mOS, median overall survival; mPFS, median progression‐free survival; NE, not evaluable; NR, not reported; Pom, pomalidomide; PVd, pomalidomide + bortezomib + dexamethasone.

^a^ Indicates if patients analyzed were refractory to only daratumumab, refractory to all CD38‐directed antibodies, triple‐refractory or penta‐refractory.

The combination of selinexor with carfilzomib and elranatamab also reported relatively long PFS and OS [31, 49]. Based on STOMP, the combination of selinexor with carfilzomib was found to have median OS and PFS at 23.7 months (95% CI: lower limit of 3.9, upper limit not evaluable) and 20.4 months (95% CI: lower limit of 20.4, upper limit not evaluable), respectively [49]. Of note, selinexor alone in STORM and MARCH yielded substantially shorter median PFS (3.7 and 2.9, respectively) and median OS (8.6 and 11.9, respectively) [46, 50]. In MagnetisMM‐3 with a median duration of follow‐up of 14.7 months, the median PFS and OS was not reached for elranatamab [31]. In the remaining studies, median OS were reported to be less than 12 months while median PFS less than 5 months.

### 3.5 Quality Assessment

All randomized controlled trials included in our review were found to have low risk of bias. For the remaining studies, the lack of a comparator arm recruited from the same population or individual‐level variables inherently prevented adjustment for confounding, leading to a critical level of risk of bias. As a result, although the level of risk of bias for other domains were considered to be low, the overall risk of bias for the nonrandomized controlled trials were still at a critical level. Traffic light and summary plots reflecting the quality of studies in our review can be found in Supporting Information S5.

## 4 Discussion

In our systematic review of clinical trials, we found that among patients with daratumumab‐refractory multiple myeloma, CAR‐T cell therapy demonstrated the highest ORR and considerably longer median OS and PFS compared to other therapeutic classes. The few randomized controlled trials that were eligible for our review confirmed this observation, in which ide‐cel and cilta‐cel both showed superior efficacy over the control arms among patients with daratumumab‐refractory disease in KarMMa‐3 and CARTITUDE‐4, respectively, with half or less the risk of progression compared to the standard of care. Promising results were also observed for elranatamab, teclistamab, ABBV‐383 (an anti‐BCMA BiTE), talquetamab and the combination of selinexor with carfilzomib.

In the current NCCN guidelines, although no specific recommendations are provided for patients who are refractory to daratumumab or CD38‐directed agents, cilta‐cel, ide‐cel, elranatamab, talquetamab, and teclistamab are listed as preferred regimens for patients who have received at least four prior lines of therapy, including an anti‐CD38 monoclonal antibody [11]. Findings from our current review supports the use of these agents in the daratumumab‐refractory population, among which CAR‐T cell therapy has emerged as the therapeutic class with the highest response rates and considerably extended survival. Interestingly, patients enrolled in studies of CAR T‐cell therapy were also younger compared to those of BiTEs and ADCs, potentially indicating a tendency to select relatively younger patients for CAR T‐cell therapy, likely out of considerations of treatment‐related toxicity. However, it should be noted that access to CAR‐T cell therapy remains limited. While a high cost is expected and inevitable with novel agents in multiple myeloma, access to CAR‐T cell therapy is further impeded by complex logistics and manufacturing bottlenecks [52]. Less than 5% of healthcare centers in the United States offer CAR‐T cell therapy [53]. As a result, a large proportion of patients eligible for CAR‐T cell therapy, including patients who have received multiple lines of treatment for multiple myeloma and who are refractory to daratumumab, will require alternative treatment options.

Of note, many of the treatment options found to yield high treatment response rates and extended survival outcomes for patients with daratumumab‐refractory disease share a common therapeutic target, which is BCMA. BCMA has been found to be overexpressed and activated in multiple myeloma based on both preclinical models and human data [54]. Existing therapeutic agents in multiple myeloma that target BCMA include ADCs, BiTEs, and CAR‐T cell therapy. BCMA‐directed treatments have previously demonstrated high antimyeloma activity and durable responses in the wider multiple myeloma patient population [54]. While our findings indicate high response rates among patients with daratumumab‐refractory disease as well, this could be due to the lack of prior exposure to BCMA‐directed drugs given the relatively recent approval of these agents and further studies are needed to confirm these observations. Additionally, several agents, such as melflufen and AMG 224, were included in our review as the studies met eligibility criteria but have been withdrawn from the market or have ceased development as treatment outcomes from the clinical trials fell short of expectations. Nevertheless, inclusion of data of these agents can serve as a benchmark for other treatments in the developmental pipeline with similar mechanisms or targets.

It should be noted that most of the studies included in our review were phase I or II trials with single‐arm designs and small samples of daratumumab‐refractory patients. Most early phase studies do not report long‐term outcomes as the focus is on safety, dosing, and tolerability. As a result, our recommendations rely on surrogate outcomes such as ORR. Without an active comparator arm and randomization, the incremental effect of most interventions over the standard of care could not be evaluated, limiting the interpretability of response rates and survival outcomes and reducing the strength of evidence to indicate if a novel agent is beneficial for patients with daratumumab‐refractory disease compared to existing options. Future randomized controlled trials are crucial to directly compare the efficacy of different BCMA‐targeting antibodies and immunotherapies and generate evidence to identify the most effective treatment options among them. The relatively small sample sizes negatively affect internal validity and increases the uncertainty of study findings. For example, although the combination of selinexor and carfilzomib showed impressive survival outcomes in STOMP, this was among 12 patients with triple refractory disease and will require additional evidence and data before a recommendation can be made. Data from early phase single‐arm trials are often used for accelerated approval of drugs to treat multiple myeloma and other cancers in general. While this improves access to novel agents, improvement in surrogate measures do not always translate into long‐term enhancement in quality of life or survival benefits [55].

A limitation of the current review is the inconsistency of the study population analyzed. We included any study which reported efficacy data of new treatments for multiple myeloma among patients with daratumumab‐refractory disease. However, some studies presented only data for patients who were triple‐ or penta‐refractory, who are also refractory to PIs and IMiDs besides daratumumab. Compared to patients who are only refractory to daratumumab, patients who are triple‐ or penta‐refractory would have disease that is harder to treat and potentially less responsive to novel agents. Nevertheless, in studies reporting on patients specifically refractory to daratumumab, a substantial proportion of patients were also refractory to PIs and/or IMiDs, suggesting that only a small number of patients had disease that was nonresponsive to daratumumab alone, likely since CD38‐directed antibodies were available much later than PIs and IMiDs. A few studies consisted of study populations that were largely refractory to anti‐CD38 agents, such as those of PHE885 and GC012F (both BCMA‐directed CAR T‐cell therapies), but failed to meet our eligibility criteria as no findings were reported for patients with daratumumab‐refractory disease. For studies that reported daratumumab‐refractory patients in a subgroup analysis, patient characteristics of the subgroups were also almost always not reported (except for HORIZON); hence, an accurate description of the patients of interest could not be obtained. Additionally, due to the lack of comparator arms and heterogeneity in the inclusion and exclusion criteria, it was not feasible to carry out a quantitative synthesis of our findings. As such, the current review is a qualitative analysis of treatment options to illustrate trends in response rates and survival outcomes associated with different therapeutic classes for patients with daratumumab‐refractory multiple myeloma. Our review also considers solely evidence on efficacy. Past studies have found that when considering treatment options for multiple myeloma, some patients may weigh other attributes, such as treatment cost, duration, and associated side effects, with similar or higher degrees of importance [56, 57]. Long‐term toxicities, such as myelodysplastic syndrome, prolonged cytopenia, and neurotoxicity, have been reported with CAR T‐cell therapies but are not usually observed with bispecific antibodies. The impact of long‐term toxicities on patient outcomes following different treatment choices also needs to be studied prospectively [58, 59].

In conclusion, findings from our review indicated the highest response rates and relatively improved survival outcomes among patients with daratumumab‐refractory multiple myeloma who received CAR‐T cell therapy compared to other therapeutic classes. Other options with potentially promising efficacy include monoclonal antibodies such as elranatamab, teclistamab, and talquetamab. With the caveat that most of these therapies have only been investigated over a short posttrial follow‐up, targeting BCMA effectively remains a huge focus of drug development in multiple myeloma. Nevertheless, more data on these treatment options, particularly on adverse events and the impact on quality of life in the long term is necessary to ensure that treatment selection is holistic and considers all treatment attributes that are important to patients. Given that the majority of eligible trials are single‐arm and lack long‐term outcomes, additional evidence of higher quality is needed before strong recommendations can be made. A head‐to‐head comparison of novel agents will be useful to generate data, not only on efficacy but also on the safety and long‐term impact of treatment options that are available for this patient population.

## Author Contributions

Conceptualization: C.J.T., A.G., and N.C. Methodology: C.J.T. and N.C. Data curation: C.J.T., D.K., and N.K. Investigation: C.J.T., D.K., N.K., A.G., and N.C. Formal analysis: C.J.T., D.K., N.K., A.G., and N.C. Supervision: A.G. and N.C. Resources: N.C. Writing (original draft): C.J.T., D.K., and A.G. Writing (review and editing): C.J.T., D.K., N.K., A.G., and N.C.

## Ethics Statement

The authors have nothing to report.

## Conflicts of Interest

A.G. has received fees from Sanofi for consulting. Other authors declare no conflicts of interest.

## Supporting information


Appendix S1.

## Data Availability

The authors have nothing to report.
