# Supplementary material for: Treatment of Multiple Myeloma in Patients Refractory to Daratumumab/Anti‐CD38 Monoclonal Antibodies: A Systematic Review
Source: Cancer Med. 2025 Mar 7;14(5):e70585. doi: 10.1002/cam4.70585 (PMC11887125; doi:10.1002/cam4.70585)

**Supplementary Material 1.** Deviations from review protocol

| Original protocol | Deviation | Rationale |
| --- | --- | --- |
| **Risk of bias assessment**  For quality assessment of single arm trials, not all domains will be relevant and no overall risk of bias will be evaluated. Instead, the level of bias for each domain will be reported separately. | The level of risk of bias for each domain was reported and the overall risk of bias will be determined based on the risk of bias in each domain. | Given that no comparator arm was available in single arm trials, the individual domains were evaluated by considering if bias in each individual domain can be addressed if an external control arm was available. This will allow quality assessment in each domain and subsequently an overall risk of bias based on each domain. |

**Supplementary Material 2.** Search syntax

**Medline (via PubMed)**

| # | Search term | Yield |
| --- | --- | --- |
| 1 | "Multiple Myeloma"[Mesh] | 47939 |
| 2 | (myeloma[Text Word]) OR (kahler[Text Word]) | 71505 |
| 3 | #1 OR #2 | 72030 |
| 4 | refractor*[Text Word] | 166246 |
| 5 | #3 AND #4 | 4926 |
| 6 | #5, FILTER: Clinical trial | 918 |
| 7 | #6, FILTER: from 2015-2023 | 493 |

**Embase (via OVID)**

| # | Search term | Yield |
| --- | --- | --- |
| 1 | 'multiple myeloma'/exp | 102296 |
| 2 | 'kahler*':ti,ab,ok,cl,de,kw OR 'myeloma*':ti,ab,ok,cl,de,kw | 133605 |
| 3 | 1 OR 2 | 133605 |
| 4 | 'refractory tumor'/exp | 26527 |
| 5 | 3 AND 4 | 381 |
| 6 | 5, FILTER: Clinical studies, EMBASE, 2015-2023 | 66 |

**Cochrane CENTRAL**

| # | Search term | Yield |
| --- | --- | --- |
| 1 | MeSH descriptor: [Multiple Myeloma] explode all trees | 2782 |
| 2 | (multiple myeloma OR myeloma* OR kahler):ti,ab,kw (Word variations have been searched) | 6577 |
| 3 | (refract*):ti,ab,kw (Word variations have been searched) | 28028 |
| 4 | (#1 OR #2) AND (#3) | 1139 |
|  | with Publication Year from 2015 to 2023, in Trials |  |

**Supplementary Material 3.** Papers that appeared to meet eligibility criteria but were excluded

| **Examples of papers that appeared to meet eligibility criteria but were excluded** | **Reason of exclusion** |
| --- | --- |
| Lesokhin A, LeBlanc R Dimopoulos MA, et al. Isatuximab in combination with cemiplimab in patients with relapsed/refractory multiple myeloma: a phase 1/2 study. Cancer Medicine. 2023;12(9)10254-10266.  doi: 10.1002/cam4.5753 | No distinction between daratumumab-refractory and daratumumab-exposed patients when reporting results. |
| Raje N, Berdeja J, Lin Y, et al. Anti-BCMA CAR T-Cell Therapy bb2121 in Relapsed or Refractory Multiple Myeloma. New England Journal of Medicine 2019;380(18)1726-1737.  doi: 10.1056/NEJMoa1817226 | No distinction between daratumumab-refractory and daratumumab-exposed patients when reporting results. |
| Oliver-Caldes A, Gonzalez-Calle V, Cabanas V, et al. Fractionated initial infusion and booster dose of ARI0002h, a humanised, BCMA-directed CAR T-cell therapy, for patients with relapsed or refractory multiple myeloma (CARTBCMA-HCB-01): a single-arm, multicentre, academic pilot study. The Lancet Oncology 2023;24(8)913-924.  doi: 10.1016/S1470-2045(23)00222-X | No distinction between daratumumab-refractory and daratumumab-exposed patients when reporting results. |
| Chen W, Wang Y, Qi K. Efficacy and Safety of Chimeric Antigen Receptor T-Cell Therapy for Relapsed/Refractory Immunoglobulin D Multiple Myeloma. Transplantation and Cellular Therapy 2021;27(3)e5.  doi: 10.1016/j.jtct.2020.12.017 | No distinction between daratumumab-refractory and daratumumab-exposed patients when reporting results. |
| Trudel S, Lendval N, Popat R, et al. Antibody-drug conjugate, GSK2857916, in relapsed/refractory multiple myeloma: an update on safety and efficacy from dose expansion phase I study. Blood Cancer Journal 2019;9(4):37.  doi: 10.1038/s41408-019-0196-6 | No distinction between daratumumab-refractory and daratumumab-exposed patients when reporting results. |
| Kaufman JL, Gasparetto C, Schjesvold FH, et al. Targeting BCL-2 with venetoclax and dexamethasone in patients with relapsed/refractory t(11;14) multiple myeloma. American Journal of Hematology 2021;96(4):418-427.  doi: 10.1002/ajh.26083 | No distinction between daratumumab-refractory and daratumumab-exposed patients when reporting results. |
| Derman BA, Chari A, Zonder J, et al. A phase I study of selinexor combined with weekly carfilzomib and dexamethasone in relapsed/refractory multiple myeloma. European Journal of Haematology 2023;110(5)564-570.  doi: 10.1111/ejh.13937 | No distinction between daratumumab-refractory and daratumumab-exposed patients when reporting results. |
| Schjesvold F, Palva B, Ribrag V, et al. Cobimetinib Alone and Plus Venetoclax With/Without Atezolizumab in Patients With Relapsed/Refractory Multiple Myeloma. Lymphoma, Myeloma and Leukemia 2023;23(1)e59-e70.  doi: 10.1016/j.clml.2022.10.006 | No distinction between daratumumab-refractory and daratumumab-exposued patients when reporting results. |

**Supplementary Material 4.** Characteristics of patients in included studies stratified by therapeutic class of intervention

| Trial | Median age  in years (range) | Male | ECOG | | | | HR cyto^†^ | Median time from dx in years (range) | Median LOT (range) | HSCT (auto) | Refractory to | | |
| --- | --- | --- | --- | --- | --- | --- | --- | --- | --- | --- | --- | --- | --- |
|  |  |  | 0 | 1 | 2 | 3 |  |  |  |  | PI | IMiD | Both |
| **CAR-T cell therapy** | | | | | | | | | | | | | |
| NCT04155749^19^ | 73 (66-75)/  60 (53-65)^‡^ | 75% | NR | NR | NR | NR | 90% | 6.5 (1.8-11.8) | 5 (3-16) | 58% | 100% | 100% | 100% |
| NCT04720313^20^ | 62 (44-75) | 40% | 35% | 20% | 45% | 0% | 50% | 4.6 (0.7-20.1) | 6 (3-13) | 85% | NR | NR | NR |
| UNIVERSAL^34^ | 64 (46-77) | 63% | 49% | 51% | 0% | 0% | 37% | 4.9 (0.9-26.4) | 5 (3-11) | 91% | NR | NR | 91% |
| CARTITUDE-1^35^ | 61 (56-68) | 59% | 40% | 56% | 4% | 0% | 24% | 5.9 (4.4-8.4) | 6 (4-8) | 90% | 100% | 100% | 97% |
| CARTITUDE-4^36^  Cilta-cel  SoC | 62 (27-78)  61 (35-80) | 56%  59% | 55%  57% | 45%  42% | 1%  1% | 0%  0% | 59%  63% | 3.0 (0.3-18.1)  3.4 (0.4-22.1) | 2 (1-3)  2 (1-3) | 1%  1% | NRˆ | 100%  100% | NR |
| KarMMa^37,38^ | 61 (33-78) | 59% | 45% | 53% | 2% | 0% | 35% | 6.0 (1.0-18.0) | 6 (3-16) | 94% | 91% | 98% | 89% |
| KarMMa-3^39^  Ide-cel  SoC | 63 (30-81)  63 (42-83) | 61%  60% | 47%  50% | 52%  47% | <1%  3% | 0%  0% | 42%  46% | 4.1 (0.6-21.8)  4.0 (0.7-17.7) | 3 (2-4)  3 (2-4) | 84%  86% | 74%  72% | 88%  94% | 67%  69% |
| **BCMA-directed monoclonal antibodies (including ADCs and BiTEs)** | | | | | | | | | | | | | |
| DREAMM-2^21-24^  2.5mg/kg  3.4mg/kg | 65 (60-70)  67 (61-72) | 53%  56% | NR | NR | NR | NR | 42%  47% | 5.5 (4.0-7.0)  5.1 (4.2-7.5) | 7 (3-21)  6 (3-21) | NR | 100%  100% | 100%  100% | 100%  100% |
| MagnetisMM-3^25^ | 68 (36-89) | 55% | 37% | 58% | 6% | 0% | 25% | NR | 5 (2-22) | 71% | 100% | 100% | 100% |
| NCT03933735^40^ | 68 (35-92) | 51% | 39% | 53% | 6% | 0% | 12% | 6.0 (1.0-15.0) | 4 (3-12) | 82% | NR | NR | 88% |
| NCT02561962^41^ | 65 (46-82) | 48% | 28% | 70% | 3% | 0% | 38% | NR | 7 (2-11) | 73% | 35% | 53% | 33% |
| MajesTEC-1^42,43^ | 64 (33-84) | 58% | 33% | 67% | 0% | 0% | 26% | 6.0 (0.8-22.7) | 5 (2-14) | 82% | 86% | 92% | NR |
| **Non-BCMA-directed monoclonal antibodies (including ADCs and BiTEs)** | | | | | | | | | | | | | |
| NCT03713294^26^ | 70 (42-84) | NR | NR | NR | NR | NR | 43% | NR | 4 (1-9) | 49% | NR | NR | NR |
| NCT02514668^27^ | 71 (51-84) | NR | 16% | 50% | 31% | 3% | 16% | 7.1 (1.2-19.4) | 7 (2-14) | NR | 81% | 91% | 75% |
| NCT02283775^44^ | 65 (45-85) | 57% | 32% | 64% | 4% | 0% | 21% | 6.2 (1.1-22.7) | 3 (1-8) | 66% | NR^*^ | NR^*^ | NR |
| MonumenTAL-1^45^  405mcg/kg  800mcg/ml | 62 (46-80)  64 (47-84) | 63%  48% | NR | NR | NR | NR | 11%  22% | 5.6 (1.7-19.6)  6.4 (0.8-21.3) | 6(2-14)  5 (2-17) | 90%  75% | NR | NR | NR |
| TRIMM-2^46^  (Talquetamab arm) | 63 (37-81) | NR | NR | NR | NR | NR | 18% | NR | 5 (2-16) | NR | NR | NR | NR |
| **Others** | | | | | | | | | | | | | |
| CC-220-MM-001^28,29^ | 64 (44-83) | 56% | 39% | 51% | 9% | 0% | 30% | 6..9 (1.6-24.5) | 6 (3-23) | 79% | 97% | 100% | 97% |
| NCT03374085^30^ | 67 (42-85) | 54% | 35% | 56% | 9% | 0% | 37% | 7.4 (1.1-37.0) | 6 (3-15) | 77% | 100% | 100% | 100% |
| STORM^31-33^ | 65 (40-86) | 58% | 30% | 58% | 9% | 0% | 53% | 6.6 (1.1-23.4) | 7 (3-18) | NR | 100% | 100% | 100% |
| HORIZON^47^ | 65 (35-86) | 59% | 22% | 63% | 15% | 0% | 34% | 6.2 (0.7-24.6) | 5 (2-12) | 68% | 100% | 100% | 100% |
| OCEAN^48^  Meflufen  Pomalidomide | 68 (60-72)  68 (61-72) | 57%  56% | 37%  37% | 53%  55% | 11%  8% | 0%  0 % | 34%  35% | 4.0 (2.6-6.2)  3.9 (2.5-6.2) | 3 (2-3)  3 (2-3) | 51%  48% | 66%  65% | 100%  100% | 66%  65% |
| STOMP^49^ | 70 (35-76) | 63% | 25% | 69% | 6% | 0% | 53% | 5.3 (0.4-11.3) | 4 (1-8) | 72% | 59% | 75% | 50% |
| MARCH^50^ | 60 (42-82) | 52% | 32% | 66% | 2% | 0% | 67% | 3.2 (0.2-13.4) | 5 (1-16) | 22% | 100% | 100% | 100% |
| NCT02899052^51^ | 66 (37-79) | NR | 31% | 59% | 0% | 27% | 27% | 1 (1-3) | 51% | 71% | 57% | 45% |  |

Note: All patients were refractory to daratumumab for studies in unshaded rows while patients with daratumumab-refractory disease were analyzed in subgroup analyses for studies in shaded rows. Except for HORIZON, patient characteristics of studies which reported patients with daratumumab-refractory disease in a subgroup analysis are of the full participant cohort.

Abbreviations: ADC, antibody-drug conjugate; BCMA, B-cell maturation antigen; BiTE, bispecific T-cell engager; CART, chimeric antigen receptor-T cell therapy; dx, diagnosis; ECOG PS, Eastern Cooperative Oncology Group Performance Status; HR cyto, high-risk cytogenetics; HSCT (auto), hemapoetic stem cell transplant (autologous); IMiD, immunomodulators; LOT, prior lines of therapy; NR, not reported; PI, protease inhibitors.

^†^ High-risk cytogenetics refer to del(17p), or t(4;14), or t(14;16).

^‡^ Reported separately for dose levels 100x10^6 cells and 300x10^6 cells:

ˆ Not reported for PI collectively but for specific PIs. Cilta-cel: 26% vs SoC: 23% for bortezomib, Cilta-cel: 25% vs SoC: 21% for carfilzomib, Cilta-cel: 7% vs SoC: 8% for ixazomib.

^*^ Not reported for PIs and IMiDs collectively but for specific drugs: 55% for bortezomib, 15% for carfilzomib, 87% for lenalidomide and 49% for pomalidomide.

**Supplementary Material 5.** Quality assessment of included studies

Figure S5A. Traffic light plot for randomized controlled trials assessed using ROB-2


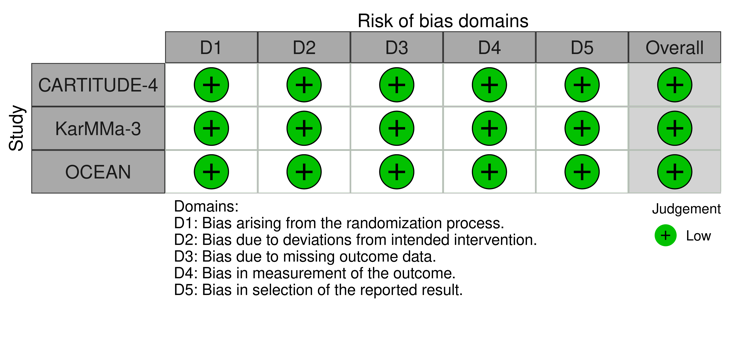


Figure S5B. Traffic light plot for non-randomized controlled trials assessed using ROBINS-I


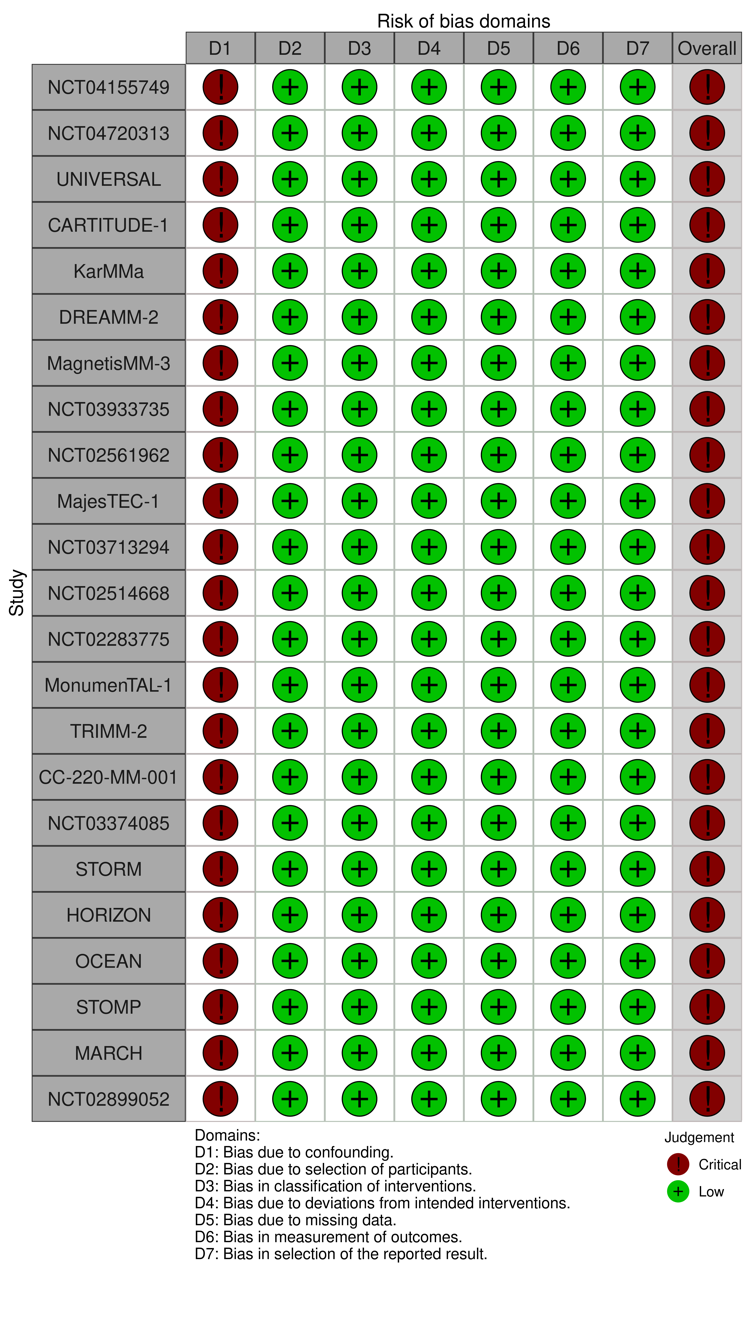


Figure S5C. Summary plot for randomized controlled trials assessed using ROB-2


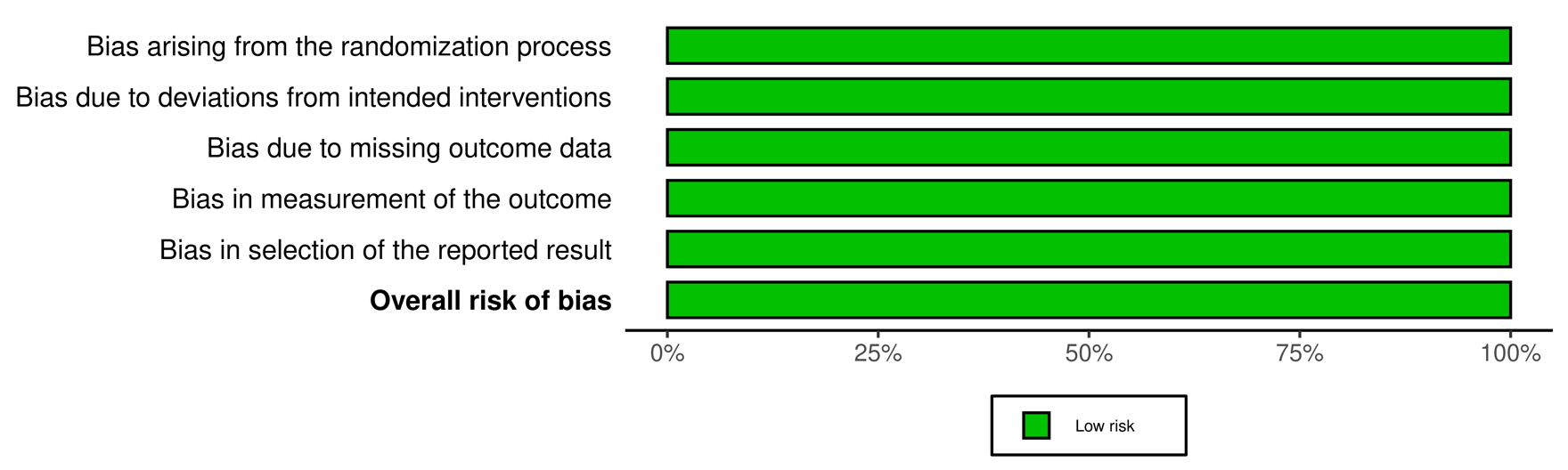


Figure S5D. Summary plot for non-randomized controlled trials assessed using ROBINS-I


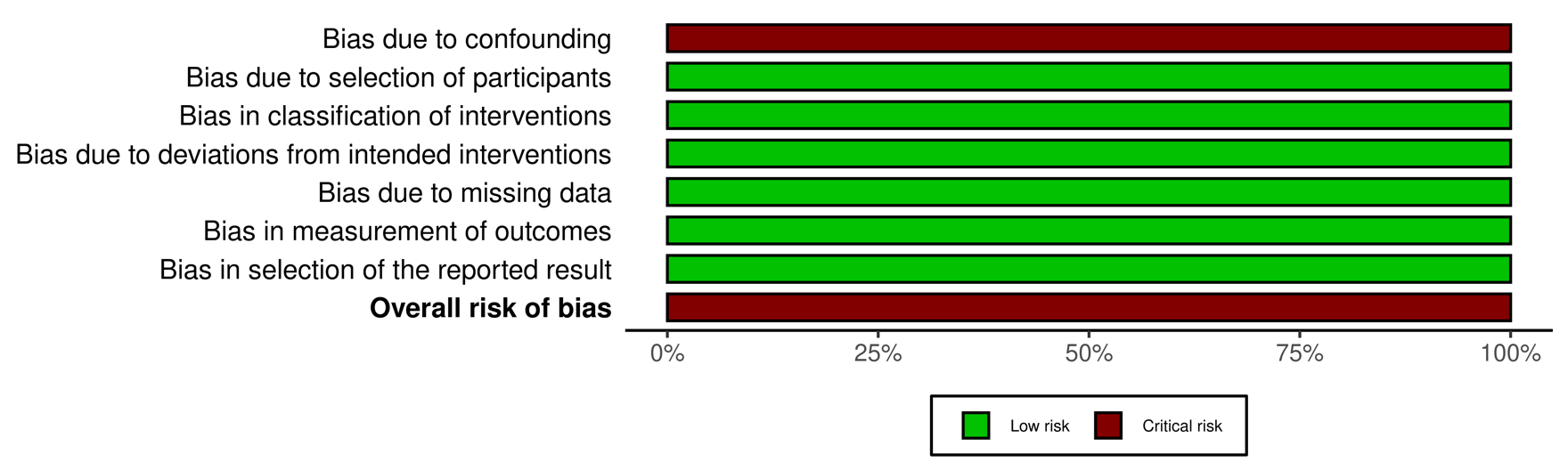

Supplement: Supplementary file 1 — Appendix S1. [file CAM4-14-e70585-s001.docx]
